# Identification of urinary metabolites that distinguish membranous lupus nephritis from proliferative lupus nephritis and focal segmental glomerulosclerosis

**DOI:** 10.1186/ar3530

**Published:** 2011-12-07

**Authors:** Lindsey E Romick-Rosendale, Hermine I Brunner, Michael R Bennett, Rina Mina, Shannen Nelson, Michelle Petri, Adnan Kiani, Prasad Devarajan, Michael A Kennedy

**Affiliations:** 1 Department of Chemistry and Biochemistry, Miami University, Oxford, OH 45056, USA; 2 Division of Rheumatology, Cincinnati Children's Hospital Medical Center, Cincinnati, OH 45229, USA; 3 Division of Nephrology and Hypertension, Cincinnati Children's Hospital Medical Center, Cincinnati, OH 45229, USA; 4 Division of Rheumatology, Department of Medicine, Johns Hopkins University School of Medicine; Baltimore, MD 21205, USA

## Abstract

**Introduction:**

Systemic lupus erythematosus (SLE or lupus) is a chronic autoimmune disease, and kidney involvement with SLE, a.k.a. lupus nephritis (LN), is a frequent and severe complication of SLE that increases patient morbidity and mortality. About 50% of patients with SLE encounter renal abnormalities which, if left untreated, can lead to end-stage renal disease. Kidney biopsy is considered the criterion standard for diagnosis and staging of LN using the International Society of Nephrology/Renal Pathology Society (ISN/RPS) classification, which was developed to help predict renal outcomes and assist with medical decision-making. However, kidney biopsy-based classification of LN is highly invasive and impractical for real-time monitoring of LN status. Here, nuclear magnetic resonance (NMR) spectroscopy-based metabolic profiling was used to identify urinary metabolites that discriminated between proliferative and pure membranous LN as defined by the ISN/RPS classification, and between LN and primary focal segmental glomerulosclerosis (FSGS).

**Methods:**

Metabolic profiling was conducted using urine samples of patients with proliferative LN without membranous features (Class III/IV; *n *= 7) or pure membranous LN (Class V; *n *= 7). Patients with primary FSGS and proteinuria (*n *= 10) served as disease controls. For each patient, demographic information and clinical data was obtained and a random urine sample collected to measure NMR spectra. Data and sample collection for patients with LN occurred around the time of kidney biopsy. Metabolic profiling analysis was done by visual inspection and principal component analysis.

**Results:**

Urinary citrate levels were 8-fold lower in Class V LN compared to Class III/IV patients, who had normal levels of urinary citrate (*P *< 0.05). Class III/IV LN patients had > 10-fold lower levels of urinary taurine compared to Class V patients, who had mostly normal levels (*P *< 0.01). Class V LN patients had normal urinary hippurate levels compared to FSGS patients, who completely lacked urinary hippurate (P < 0.001).

**Conclusions:**

This pilot study indicated differences in urinary metabolites between proliferative LN and pure membranous LN patients, and between LN and FSGS patients. If confirmed in larger studies, these urine metabolites may serve as biomarkers to help discriminate between different classes of LN, and between LN and FSGS.

## Introduction

Systemic lupus erythematosus (SLE or lupus) is a chronic autoimmune disease [1]. Kidney involvement with SLE, a.k.a. lupus nephritis (LN), is a frequent and severe complication of SLE that increases patient morbidity and mortality [2]. About 50% of patients with SLE encounter renal abnormalities which, if left untreated, can lead to end-stage renal disease [3,4].

Kidney biopsy is considered the criterion standard for diagnosis and staging of LN using the International Society of Nephrology/Renal Pathology Society (ISN/RPS) Classification [5]. This classification was developed to help predict renal outcomes and assist with medical decision-making. Treatment of patients diagnosed with ISN/RPS class III or class IV LN requires combination therapy with corticosteroids plus immunosuppressive medications [6], whereas therapeutic choices for class V LN are still under considerable debate [7]. The ISN/RPS class of a patient with LN is not static. Over time, histological features of LN may improve in response to therapy or degenerative changes can accrue. The lack of sensitive and specific non-invasive biomarkers that assist with distinguishing between various LN classes makes it virtually impossible to dynamically monitor changes in LN classes in real time. This impairs the timely initiation of therapy and impairs monitoring of treatment response.

It is particularly difficult to discriminate proliferative LN (class III/IV) from pure membranous LN (class V) clinically, as both are associated with pronounced proteinuria, and changes in blood pressure and renal function. Pronounced proteinuria is also the hallmark of primary focal segmental glomerulosclerosis (FSGS). One of the histological characteristics of FSGS is podocyte injury, which results in different degrees of proteinuria and potentially, hypoalbuminemia, that is, these clinical and histological features can also occur with active LN [8].

In the past, we and others have used proteomics to discover candidate protein biomarkers for LN [9,10]. Alternative biomarker discovery approaches include metabolomics, that is, the systematic study of small-molecule metabolite profiles or unique chemical fingerprints that are the result of specific cellular processes, and metabonomics, which can be defined as the quantitative measurement of metabolite changes in such metabolic profiles [11-13]. Nuclear magnetic resonance (NMR) finger printing is currently the method of choice for metabonomics because it provides uniform detection of equal sensitivity for all proton-containing small molecules and can provide valuable information on metabolites directly from biofluids with little sample preparation [14-16]. Metabonomics is achieved by maximum data capture through NMR spectroscopy followed by pattern recognition statistics [17].

The objective of this study was to identify urinary metabolites that discriminated between proliferative LN (class III/IV), pure membranous LN (class V), and FSGS, using NMR spectroscopy-based metabonomics. Metabolic profiles of urine samples were investigated using high-field (850 MHz) solution-state NMR spectroscopy. Two urinary metabolites, citrate and taurine, were found to accurately distinguish between class III/IV and class V LN patients. Urinary citrate levels were eight-fold lower than normal in class V compared with class III/IV LN patients, who had normal levels of urinary citrate. Also, class III/IV LN patients had more than 10-fold lower than normal levels of urinary taurine compared with class V patients, who had mostly normal levels of urinary taurine. Finally, urinary hippurate levels accurately distinguished between class V patients, who had normal levels of urinary taurine, in comparison with FSGS patients, who completely lacked urinary hippurate.

## Materials and methods

### Patients and samples

All research was conducted in compliance with the Helsinki Declaration. Informed consent was obtained from all enrolled patients. The study was approved by the institutional review boards of both the Johns Hopkins Hospital and the Cincinnati Children's Hospital Medical Center. Children and adults diagnosed with SLE [1] who required a kidney biopsy as part of standard of care therapy were eligible for inclusion in this study if a random spot urine sample was available that was collected within 60 days of the kidney biopsy. On the day of the urine sample collection, information about patient demographics, medications, and disease activity was collected. Key laboratory measures were obtained, including complement C3 and C4 levels, anti-dsDNA antibodies (present/absent), amount of proteinuria as estimated by the protein to creatinine ratio (P/C ratio) in a random or 24-hour urine sample, serum creatinine, and glomerular filtration rate (GFR) as estimated by age-appropriate calculation of the creatinine clearance [18,19]. For SLE patients to be included in the study, they had to have undergone kidney biopsy, found to have either class III or IV LN without membranous features (class III/IV) or pure membranous class V LN as per the ISN/RPS classification [5], had an available stored urine sample collected within 60 days of a kidney biopsy, and signed the informed consent form.

The histological characteristics of each kidney biopsy, as per report from the local pathologists, were reviewed in a blinded fashion by one expert nephropathologist, as per the ISN/RPS classification [5]. The following histological features reflective of active inflammation with LN were recorded: mesangial proliferation, endocapillary karyorrhexis (also: fibrinoid necrosis); cellular crescents; capillary proliferation, subendothelial deposits identifiable by light microscopy (also: wire-loops). We also noted features representing LN chronicity or degenerative damage. These included glomerular sclerosis (segmental or global), fibrosis including fibrous adhesions and fibrous crescents, as well as tubular atrophy. The results of these classifications are summarized in supplementary Table S1 [see Additional data file 1].

Almost all LN studies employ a previously developed scoring system to quantify the amount of overall LN activity and overall LN chronicity present in kidney biopsy specimens [20]. The features of activity and chronicity listed above were categorized as follows: 0 (no lesions), 1 (lesions in up to 25% of glomeruli), 2 (lesions in 25 to 50% of glomeruli) or 3 (lesions in > 50% of glomeruli). Using these numeric values, a Biopsy Activity Index (AI) score (range 0 to 24) and a Biopsy Chronicity Index (CI) score (range 0 to 12) can be calculated, where higher scores represent higher LN activity or chronicity, respectively. The ISN/RPS classification, the AI and the CI have all been validated for use in adults and children with LN [21,22]. Risk factors for poor LN outcome include AI scores of seven or higher and CI scores of four or higher [21,23-30]. The AI and CI scores of the patients are also listed in supplementary Table S1 [see Additional data file 1].

Epimembranous deposits, although not included in the AI or the CI scores, were also recorded. Depending on the findings of active inflammation, and chronic changes observed on kidney biopsy, LN is classified in six categories. Pronounced predominance of epimembranous deposits is compatible with class V of LN.

For patients with LN, key laboratory measures were recorded, including complement C3 levels, anti-dsDNA antibodies (present/absent), amount of proteinuria as estimated by the P/C ratio in a random or 24-hour urine sample, serum creatinine, and GFR as estimated by age-appropriate calculation of the creatinine clearance [18,19]. The renal domain score of the Systemic Lupus Erythematosus Disease Activity Index (SLEDAI-R; range 0 to 16; 0 = inactive LN) served as the clinical measure of LN activity [31]. The Systemic Lupus International Collaborating Clinics/American College of Rheumatology Damage Index items addressing kidney damage (SDI-R; range 0 to 3; 0 = no LN damage) were recorded as a clinical measure of kidney damage with LN [32]. The results of these laboratory measurements are summarized in supplementary Table S2 [see Additional data file 1].

Ten patients with biopsy-proven primary FSGS and proteinuria served as a disease control group. For controls with FSGS, data and urine samples were collected during visits to the pediatric nephrology clinics.

The demographics and kidney status of all patients included in the study are summarized in Table 1.

**Table 1:** Patient demographics, medications and renal status at the time of the urine collection.

		Focal segmental glomerulosclerosis		Proliferative LN (class III or IV)		Pure membranous LN (class V)	
		n of 10 (%)	Median (Range)	n of 7 (%)	Median (Range)	n of 7 (%)	Median (Range)
**Females**		3 (33%)		4/7 (57%)		5/7 (71%)	
**Race**	Black	2/10 (20%)		3/7 (42%)		3/7 (42%)	
	White	5/10 (50%)		2/7 (29%)		2/7 (29%)	
	Other	3/10 (30%)*		2/7 (29%)†		3/7 (43%)**	
**Medications**	Oral prednisone	4/10 (40%)		7/7 (100%)		4/7 (57%)	
	Mycophenolate mofetil	5/10 (50%)		3/7 (43%)		5/7 (71%)	
	Cyclophosphamide	-		2/7 (29%)		1/7 (14%)	
	Angiotensin blocking agent	9/10 (90%)		2/7 (29%)		6/7 (86%)	
**Kidney Status**	GFR < 60 ml/min/m^2^	4/10 (40%)		1/7 (14%)		1/7 (14%)	
	Protein: creatinine ratio > 0.5	5/5 (100%)		7/7 (100%)		7/7 (100%)	
	Renal SDI score > 0¥	-		0/3		1/4 (25%)	
	Renal SLEDAI score^‡^	-			4 (0-16)		8 (4-12)
	Presence of anti double-stranded-dsDNA	-		7/7 (100%)		4/7 (67%)	

‡ Systemic Lupus Erythematosus Disease activity Index (SLEDAI) - renal component score; ¥ Systemic Lupus International Collaborating Clinics/American College of Rheumatology Damage Index - renal component score; *American Indian 1, Asian 1, Mixed racial 1; † Asian 1, Mixed racial 1; ** Asian 1, Unknown

### Metabolic profiling

#### Preparation of urine samples for NMR analysis

Urine samples were stored at -80°C after collection and thawed on ice prior to preparation for NMR analysis. A 1 ml aliquot of each sample was centrifuged for 10 minutes at 2655 × g, and then 350 μl of clear urine was pipetted into a 1.5 ml microcentrifuge tube. A volume of 350 ml of buffer (300 mM KH_2_PO_4_, 2 mM NaN_3_, 0.2% trimethylsilyl propionate (TSP) in 20% D_2_O, pH 7.4) was added to each urine sample. A volume of 600 μl of the urine/buffer mixture was then pipetted into a 5 mm NMR tube (Norell ST500-7, Norell, Inc., Landisville, NJ USA).

#### NMR data collection and processing

All NMR experiments were carried out on a Bruker Avance™ III spectrometer (Bruker Biospin, Rheinstetten, Germany) operating at 850.10 MHz ^1^H frequency and equipped with a room temperature 5 mm triple resonance probe with inverse detection and controlled by TopSpin 2.1.4 (Bruker Biospin, Rheinstetten, Germany). All experiments were conducted at 298 K. All data were collected using a spectral width of 20.0 ppm. Three ^1^H NMR experiments, optimized by Bruker (Bruker BioSpin, Billerica, MA, USA) for use with metabonomic studies, were run on all samples: a standard 1D presaturation (zgpr), a 1D first increment of a nuclear Overhauser effect spectroscopy (NOESY; noesygppr1d) experiment, and a CPMG (cpmgpr1d) experiment. All experiments included presaturation of the water peak. The transmitter offset frequency (O1) was set to 4002.80 Hz to obtain optimal water suppression. The 90° pulse width was determined for every sample using the automatic pulse calculation feature in TopSpin. All pulse widths were between 13 and 16 μs. Water suppression was achieved by irradiation of the water peak during a recycle delay of 4.0s with a pulse power level of 55.92 dB.

One-dimensional zgpr ^1^H NMR spectra were acquired using two transients and two dummy scans, 65 K points per spectrum giving an acquisition time of 1.92 s, -0.01 Hz of exponential line broadening, and a recycle delay of 4 seconds. Once the zgpr spectrum was determined to be of acceptable quality, based on the line width (< 1 Hz) and line shape (resolved C13 satellites) of the TSP internal standard, the other two experiments were run. The first increment of the 1D NOESY experiment was collected using eight transients with four dummy scans, 65 K points per spectrum giving an acquisition time of 1.92 seconds, a mixing time of 10 ms, and apodized using a Gaussian line broadening parameter of 0.01, and a 4 seconds recycle delay. The CPMG experiment was collected in order to eliminate any broad peaks present in the spectrum. The CPMG experiment used 64 transients with four dummy scans, 65 K points per spectrum giving an acquisition time of 1.87 seconds, a T_2 _filter loop of 128 with an echo time of 1 ms, apodized using -0.01 Hz of exponential line broadening, and a 4 seconds recycle delay.

All NMR spectra were phased, baseline corrected, and corrected for chemical shift registration relative to TSP in TopSpin 2.1.1 (Bruker Biospin, Billerica, MA, USA).

#### Box and whisker plot analysis

Box and whisker plots were generated in excel using a template provided by Vertex42 LLC

#### Principal component analysis

The data were subjected to multivariate statistical analysis using AMIX software version 3.9.7 (Bruker Biospin, Billerica, MA, USA). All NMR spectra were normalized to total intensity prior to principal component analysis (PCA). NMR spectra were binned into 0.03 ppm-wide buckets, using simple rectangular bucketing, over the region of δ10.0 to 0.2 ppm. The region of δ 4.75 to 5.0 was excluded from the analysis to avoid effects of imperfect water suppression. Buckets with variances less than 5% were also excluded from PCA.

Unsupervised PCA was performed without consideration of group information (class III/IV; class V, FSGS). The algorithm employed to calculate the principal components (PC) is discussed by Rousseau *et al*. [33]. As is commonly done with metabolomic data, visualization of the data was accomplished by inspection of the PC scores plots and loadings plots.

#### Statistical significance analysis of NMR data

Statistical significance analysis was performed for the comparison of class III/IV and class V LN, as well as the comparison of class V LN and FSGS, using AMIX 3.9.7 (Bruker Biospin, Rheinstetten, Germany), as outlined by Goodpaster *et al*. [34]. A critical value of alpha of 0.05 was selected to ensure no greater than a 5% false positive rate. In order to correct for multiple simultaneous testing, a Bonferroni correction was applied to the critical value to ensure a constant family-wise false positive rate [34]. The Bonferroni corrected critical value was calculated by dividing alpha by the number of buckets used in the PCA, resulting in a stringent *P *value threshold for determination of statistically significant changes in resonance intensities between groups being compared. The number of buckets used for statistical significance analysis was determined by the number remaining after omitting buckets that contained less than 5% variance and after omitting buckets in the excluded regions. A change in bucket intensity between groups was determined to be statistically significant if its *P *value was less than the Bonferroni corrected critical value.

#### Mahalanobis distance and F-value calculations

Mahalanobis distance calculations and F-value calculations were performed in MatLab as described by Goodpaster *et al*. [35]. Critical F-values were calculated using a critical F-value calculator [36].

#### Identification of metabolites

Experimental NMR spectra obtained from study samples were compared with spectra of known metabolites using the ChenomX NMR Suite (ChenomX Inc., Edmonton, Alberta, Canada). The ChenomX database was used to filter for resonance frequencies at chemical shifts corresponding to those identified as outliers by visual comparison as well as in the loadings plot. Spectra present in the ChenomX database were examined to verify if the pattern of peaks matched those observed in the experimental data.

## Results and discussion

### Patients

A total of seven patients with proliferative LN without membranous features (class III/IV), seven patients with pure membranous LN (class V), and 10 disease controls with primary FSGS were included in the study.

### NMR metabonomics data analysis

Due to slight variations in the pH of the urine samples, the NMR peaks of some metabolites experienced pH-dependent chemical shifts, making it difficult to identify variances for these peaks by PCA using standard rectangular bucketing. Therefore, all spectra were also visually inspected to validate NMR resonances that were potentially changing between groups, and then these peaks were locally aligned to enable reliable *P *score calculations.

**Figure 1:**
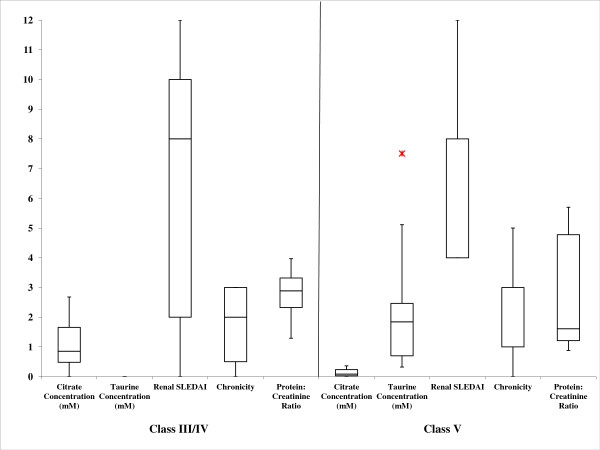
**Box and whisker plots**. The plots shown are comparing citrate concentration (mM), taurine concentration (mM), SLE Disease Activity Index (SLEDAI), chronicity index (CI), and urine creatinine (mg/mL) for class III/IV versus class V LN patients.

Visual inspection of the NMR spectra of class III/IV and class V LN patients led to the identification of one metabolite, citrate, that had an eight-fold higher urinary concentration (Table 2) (*P *score = 0.0477) in class III/IV LN patients (1.11 ± 0.97 mM) compared with class V patients (0.14 ± 0.15 mM). The concentration of citrate in the class III/IV group fell into the normal range in human urine [37] whereas the citrate levels in the class V group were at the lower limit of published values [37]. A receiver operator characteristic (ROC) analysis indicated the citrate had 100% specificity at 86% sensitivity and an overall 88% accuracy [see Additional data file 1] for distinguishing between class III/IV and class V LN patients. Urinary citrate levels were also compared with the SLEDAI-R, CI, and P/C ratio (Figure 1). The comparisons shown in Figure 1 further indicated that there was a strong correlation between disease class and citrate levels; however, no correlation was observed between class and SLEDAI, CI, or P/C ratio between class III/IV and class V LN patients. Complete summaries of measured patient indices, including others not presented in Figure 1, are presented in Tables S1 and S2 of the Supplementary Material.

**Table 2:** Concentrations and fold-changes of urinary metabolites measured in Class III/IV, Class V LN and FSGS patients.

Citrate	Class IV (mM)	Class V (mM)	Fold change
	0.59	0.15	
	2.16	0.32	
	2.68	0	
	0.85	0.36	
	1.15	0.055	
	0	0	
	0.37	0.083	
ave (std)	1.11 (0.97)	0.14 (0.15)	8.04
			
Taurine	Class IV (mM)	Class V (mM)	Fold change
	0	1.84	
	0	7.51	
	0	2.32	
	0	0.32	
	0	2.61	
	0	0.85	
	0	0.55	
ave (std)	0	2.29 (2.47)	> 10
			
Hippurate	Class V	FSGS (mM)	Fold change
	1.79	0	
	0.70	0	
	0.55	0	
	0.47	0	
	1.49	0	
	1.66	0	
	1.89	0	
		0	
		0	
		0	
ave (std)	1.22 (0.62)	0	> 10
			

**Figure 2:**
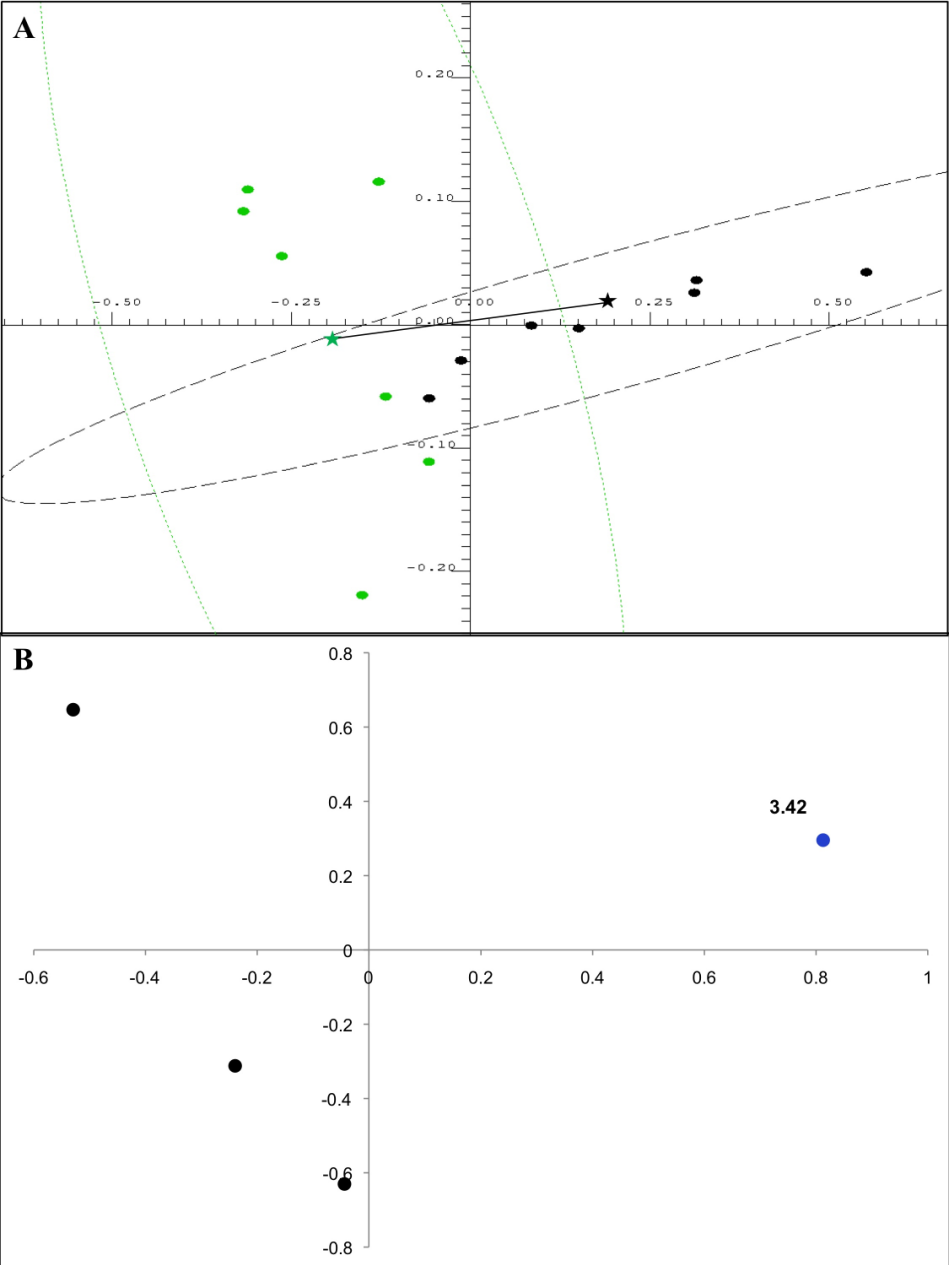
**Principal component analysis of urine samples from patients with class III/IV LN and class V LN**. **(a) **Two-dimensional principal component analysis scores plot of urine samples from patients with class III/IV LN (green) and class V LN (black) for peaks in the region from δ 3.40 to 4.50 ppm calculated using the first two principal components. Each point in the scores plot represents the NMR spectrum of an individual patient projected onto the two-dimensional space defined by the first two principal components. The dashed lines encircling the points define the 95% confidence intervals for each group. The color-matched stars indicate the centroid of each group and the line connecting the stars represents the Mahalanobis distance between the group centroids. **(b) **The loadings plot corresponding to the scores plot shown in Figure 1a. The labeled bucket (point) corresponds to the triplet belonging to taurine in the ^1^H NMR spectra. The coordinates of each point indicate the PC loadings for that bucket, and represent how strongly that bucket is weighted in the eigenvector defining either the first or second principal component. The loadings plot points are heat map color-coded according to bucket *P *value: Black (> 1.25 × 10^-2^), Blue (1.25 × 10^-2^-10^-5^). The Bonferroni corrected α-value was 0.0125.

Visual comparison of the NMR spectra of urine samples of patients with class III/IV versus class V LN also revealed higher concentrations (> 10 fold) of taurine in class V LN (2.29 ± 2.47 mM) compared with class III/IV LN patients, which lacked taurine altogether (Table 2) (*P *score = 0.00141). Taurine is normally present urine at levels in the range from 50 to 750 μM [38]. Therefore, the complete absence of taurine in class III/IV patients indicated a renal pathology that was distinct from class V patients who appeared to have mostly normal levels of taurine, with the exception of one patient who had more than five times the normal amount of taurine in the urine (Table 2). Discrimination of the two groups (class III/IV versus class V) based on urinary taurine levels was confirmed by PCA by restricting the spectral analysis just to the region of the ^1^H NMR spectra that contained the taurine-specific triplet at δ 3.425 ppm (Figure 2). Inspection of the PC scores plot of these samples showed a significant separation of the class III/IV from the class V populations, primarily based on higher levels of taurine in the urine of patients with class V LN (Figure 2a). The magnitude of the cluster separation was quantified by calculating the Mahalanobis distance between the cluster centroids and the statistical significance of the cluster separation was evaluated by calculating the F-value and comparing it with the critical F-value (Figure 2a). The Mahalanobis distance of 2.282 between class III/IV and class V LN group centroids and the corresponding F-value of 8.353 (critical F-value 3.982) indicated a statistically significant separation of the two groups. The PC loadings plot corresponding to the PC scores plot shown in Figure 2a is shown in Figure 2b. The difference in the mean intensities of the bucket at δ 3.425 ppm, which corresponded to taurine, was found to be statistically significant with a *P *value of 0.00151.

ROC curve analysis indicated that taurine was a perfect predictor for discrimination between class III/IV and class V LN patients with 100% specificity, 100% sensitivity and 100% accuracy [see Additional data file 1]. Urinary taurine levels were also compared to SLEDAI, CI, and P/C ratio (Figure 1). As indicated above, the data failed to reveal any correlation between LN class and SLEDAI, CI, or urinary P/C ratios; however, a strong correlation between class III/IV and class V LN and was observed with a complete absence of urinary taurine in class III/IV patients compared with mostly normal levels of taurine in class V patients.

**Figure 3:**
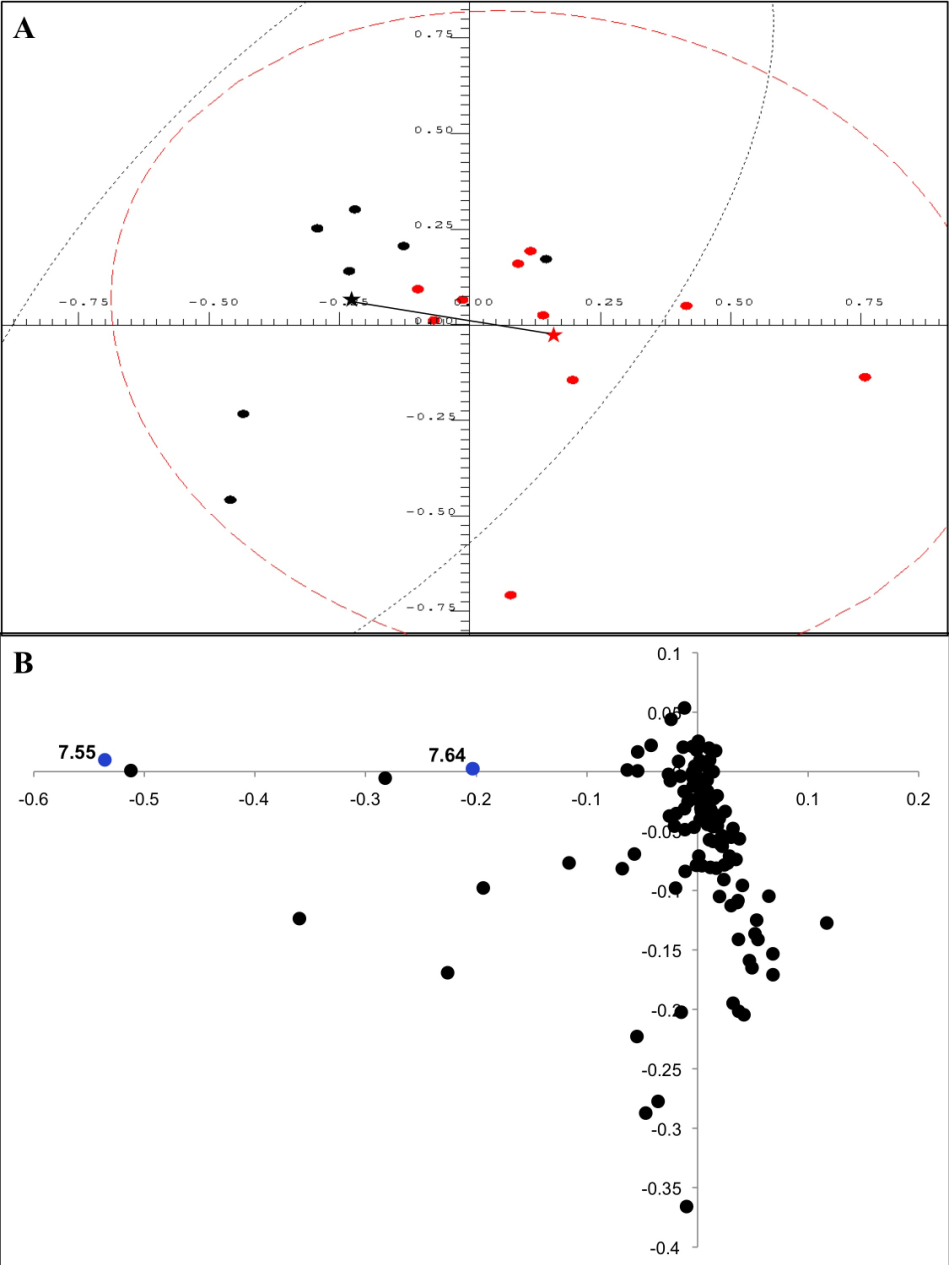
**Principal component analysis of urine samples from class V LN patients and focal segmental glomerulosclerosis patients**. **(a) **Two-dimensional principal component analysis scores plot of urine samples from patients with class V LN patients (black) and focal segmental glomerulosclerosis patients (red) using the first two principal components. The dashed lines encircling the points define the 95% confidence intervals for each group. The color-matched stars indicate the centroid of each group and the line connecting the stars represents the Mahalanobis distance between the group centroids. **(b) **The loadings plot corresponding to the scores plot in Figure 2a. The buckets shown are in the region from δ 0.02 to 10.0 ppm. The loadings plot is heat map color-coded according to bucket *P *values: Black (> 1.730 × 10^-4^), Blue 1.730 × 10^-4^-10^-5^). The Bonferroni corrected α-value was 1.730 × 10^-4^.

**Figure 4:**
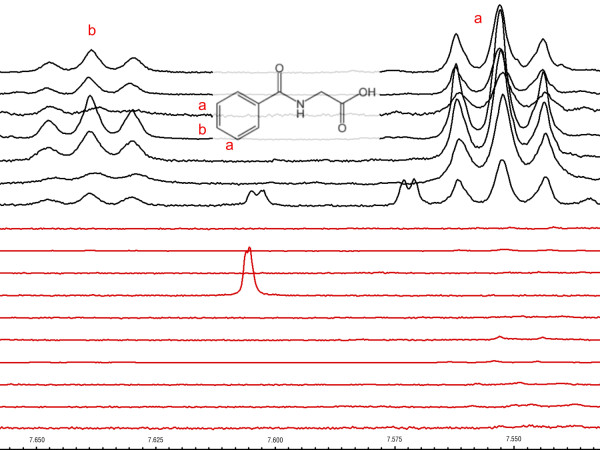
**NMR urine spectra in the region from 7.650 to 7.550 ppm of class V LN patients (black) and focal segmental glomerulosclerosis patients (red)**. The triplets at δ7.64 and δ7.55 belong to the metabolite hippurate, as indicated in the inset.

Unsupervised PCA was also carried out on normalized ^1^H NMR spectra obtained from urine samples from Class V LN and FSGS patients. Inspection of the PC scores plot showed separation of the class V from the FSGS group based on the levels of hippurate, which were in the normal range found in human urine [37] in the class V LN group (1.22 ± 0.62 mM), compared with the FSGS group, which completely lacked hippurate (Figure 3a; Table 2). The Mahalanobis distance between the cluster centroids of the class V and FSGS groups was 1.781, with a corresponding F-value of 6.096 (critical F-value 3.739) indicating a statistically significant separation of these groups. NMR spectra of patients with class V LN or FSGS were visually compared to validate the differences in hippurate concentrations indicated by PCA. Figure 4 shows a region of the NMR spectrum that contains two triplets unique to hippurate located at δ 7.64 and δ 7.55 ppm confirming that hippurate was present in the class V group but completely absent in the FSGS group. The differences in the mean bucket intensities at δ 7.55 ppm and δ 7.64 between the two groups (> 10-fold change) were found to be significant: δ 7.55 *P *value = 0.000171, and δ 7.64 *P *value = 0.000379, respectively. ROC curve analysis indicated that hippurate was a perfect discriminator for distinguishing between class V LN and FSGS patients with 100% specificity, 100% sensitivity, and 100% accuracy [see Additional data file 1]. Interestingly, the complete lack of hippurate in the FSGS group indicated a unique pathology in FSGS compared with LN that leads to depletion of the normal urinary levels of hippurate.

Kidney biopsies are currently required to distinguish between different classes of LN, and between LN and other glomerular disorders, based on characteristic histological features. Obtaining kidney biopsies is invasive, and repetitive performance to guide day-to-day medical decisions is not practical. Although diagnostic, kidney biopsies are not suited to pinpoint altered metabolic processes or biological pathways involved in LN, which if detected, could lead to the identification of novel therapeutic targets. As the kidneys filter and reabsorb metabolites to maintain a metabolic equilibrium, existence of renal pathologies can impair the filtration of small metabolites through the glomerulus and their subsequent re-absorption in the renal tubules leading to changes in metabolic profiles [39]. Using NMR-spectroscopy, we found the metabolites taurine, citrate, and hippurate differentially excreted in the urine of patients with proliferative LN, membranous LN, and FSGS.

Comparison of the metabolic profiles of class III/IV LN versus class V LN patients indicated that class III/IV patients had normal urinary citrate levels but low urinary taurine levels whereas class V LN patients exhibited low urinary citrate levels but elevated urinary taurine levels. Based on previous reports, citrate and taurine are both measures of tubular cell function [40]. A possible explanation for the reduced excretion of urinary citrate in the class V LN group could be the presence of metabolic acidosis, which is known to cause decreased urinary excretion of citrate in humans [41]. It is believed that patients experiencing low urinary citrate output may have renal tubular cells that are more acidotic than in the healthy normal populations [42]. The proximal tubules are responsible for the regulation of re-absorption and excretion of citrate [43,44]. The body's response to metabolic acidosis includes an increase of sodium/citrate co-transporter activity in the kidney, which causes increased citrate transport across the apical membrane into the tubule lumen [44]. Up-regulation of the co-transporter activity results in the increased re-absorption of citrate and reduced excretion of the metabolite into the urine. The cytosolic citrate metabolism also plays a key role in regulating the amount of citrate excreted into the urine. During metabolic acidosis, alterations in the enzyme ATP citrate lyase also results in decreased urinary citrate excretion [44].

Class III/IV LN patients had a striking absence of urinary taurine. Although the pathology leading to a complete absence of urinary taurine is not obvious, the body's store of taurine is known to be regulated by the kidneys and taurine is known to act as an anti-oxidant in a variety of *in vitro *and *in vivo *systems, and is used to treat renal dysfunction [45]. Therefore, it is possible that under the conditions of the most severe LN in class III/IV patients the kidneys utilize all available taurine in an attempt to manage or repair the kidney pathology.

Although the majority of patients with class V LN have normal levels of taurine, one patient had more than five times the normal amount of taurine. A possible explanation for the elevated level of taurine in the urine of this exceptional class V LN patient may be a consequence of inadequate re-uptake of taurine into the cells [46]. Taurine is excreted through both bile and urine, but its total body pool is primarily controlled by the kidneys via the renal tubules [47]. Previous studies suggest that tubular dysfunction is a risk factor of taurine deficiency [48]. Patients in renal failure often have low muscle and plasma concentrations of taurine. Although it has been suggested in the past that this was due to reduced taurine synthesis [48], our results suggest that low taurine levels are actually the result of increased urinary taurine excretion.

Increased urinary taurine may also be a result of changes in cysteine metabolism. Hypercysteinemia is associated with alterations of the sulfur metabolism and/or sulfate transport [49]. Taurine is known to play a critical role in these processes [49], and patients with class V LN may be unable to adequately cope with oxidative stress and the elimination of free radicals. Interestingly, based on animal studies, the acquisition of age-related renal fibrosis can be decreased by taurine supplementation [50], and taurine also has anti-hypertensive effects [51].

Histological scoring and quantification of proteinuria are key methods used to survey disease activity and severity in patients with LN [52,53]. Pirani *et al*. created a scoring system that is semi-quantitative [54], which was later adapted by Austin *et al*. [20] This system was developed to calculate the activity of LN (SLEDAI) by assessing six histological factors focusing on the severity of active lesions in the glomeruli, and the chronicity of the disease by evaluating four histological parameters focusing on the reversibility of LN [53]. Wallace *et al*. provides a table (Table 55-5 in the original article) that outlines the scoring strategies for both the AI and CI [55]. The degree of proteinuria is determined by measuring the P/C ratio in a 24-hour urine collection. This ratio has previously proven to be a reliable predictor of proteinuria in a study of LN patients [56]. The changes in citrate and taurine levels in the patients included in this study were plotted against the SLEDAI, CI, and P/C ratios in order to determine whether a correlation existed between any of these conventionally measured indices and biopsy-determined LN class (Figure 1). The data showed that no clear correlation existed between these conventional indices or P/C ratios and the LN class; however, our data indicated strong correlations between citrate and taurine levels and LN class. The lack of correlation between renal activity and chronicity with disease class is not surprising given that other studies have shown that when applying these indices to all World Health Organization classes of LN, rather than just diffuse proliferative LN, these indices lack an association with long-term prognosis [57,58]. The inability to relate P/C ratio to disease class is also expected because differing degrees of proteinuria are present throughout the patient population. Visual comparison of the above indices as they related to LN class further demonstrated a strong need for continued development of reliable biomarkers that allow for LN class differentiation, as was seen in the changes in the metabolites identified in this study.

Our pilot study also identified one urinary metabolite, hippurate, whose levels differentiated class V LN and FSGS patients. Specifically, class V LN patients had normal levels of urinary hippurate whereas FSGS patients completely lacked hippurate. Although the pathological link associated with a complete lack of urinary hippurate is not evident, the complete lack of urinary hippurate in FSGS patients is striking. A possible pathological cause could be related to having a distinct gut microbial biota, which has been linked to depleted excretion of hippurate in patients with Crohn's disease [59].

## Conclusions

Currently, it is difficult to distinguish between class III/IV and class V LN, and FSGS using conventionally measured indices. Diagnoses require invasive and time-consuming procedures involving biopsies and histological analyses, which make monitoring of real-time changes in the disease pathology impossible using current technologies. Ideally, one would like to develop a non-invasive and rapid biomarker-based methodology to distinguish between class III/IV and class V LN, and FSGS. Here we report putative urinary biomarkers for this purpose obtained from a pilot study. Using NMR spectroscopy, we found that the metabolites taurine, citrate, and hippurate were differentially excreted in the urine of patients with proliferative LN, membranous LN, and FSGS. Not only do these metabolites represent potential biomarkers for distinguishing classes of LN and FSGS, but consideration of the metabolic pathways involving these metabolites should lead to a better understanding of the pathology of the respective disease states. Although the small size of this pilot study limits its statistical power, it is in the realm of similar pilot studies for other diseases [60]. Nonetheless, our study has generated several hypotheses regarding the etiology of LN and FSGS and further validation of our findings is planned using an independent cohort of patients.

## Abbreviations

AI: activity index; CI: chronicity index; FSGS: focal segmental glomerulosclerosis; GFR: glomerular filtration rate; ISN/RPS: International Society of Nephrology/Renal Pathology Society; LN: lupus nephritis; NMR: nuclear magnetic resonance; NOESY: nuclear Overhauser effect spectroscopy; P/C ratio: protein to creatinine ratio; PC: principal component; PCA: principal component analysis; ROC: receiver operator curve; SLE: systemic lupus erythematosus; SLEDAI-R: Systemic Lupus Erythematosus Disease Activity Index-Range; SDI-R: Systemic Lupus International Collaborating Clinics/American College of Rheumatology Damage Index-Range; TSP: trimethylsilyl propionate.

## Competing interests

Drs. Prasad Devarajan and Brunner are holding a patent on the use of some of the renal biomarkers evaluated for this study.

## Authors' contributions

HIB was involved in the study design, data collection, and sample management as well as the interpretation of the data and the development of the manuscript. MRB, RM, SN, MP, and AK were all involved in the data collection, and sample collection and management and the development of the manuscript. PD was responsible for the study design, the interpretation of the data, and the development of the manuscript. LRR was responsible for NMR sample preparation, NMR data collection and analysis, investigation of biological interpretation of data and development of the manuscript. MAK was involved in NMR data analysis, interpretation of data and development of the manuscript. All authors read and approved the final manuscript.

## Supplementary Material

Additional file 1 **There are two additional tables included in this file, including Table S1, which provides a summary of classifications from histological analyses of kidney biopsy samples, and Table S2, which is a summary of laboratory test scores for LN patients**. The file also contains Figure S1, which includes the receiver operator characteristic curves for citrate, hippurate and taurine. Click here for file
