# Supplementary material for: Identification of urinary metabolites that distinguish membranous lupus nephritis from proliferative lupus nephritis and focal segmental glomerulosclerosis
Source: Arthritis Res Ther. 2011 Dec 7;13(6):R199. doi: 10.1186/ar3530 (PMC3334650; doi:10.1186/ar3530)
Supplement: Additional file 1 — There are two additional tables included in this file, including Table S1, which provides a summary of classifications from histological analyses of kidney biopsy samples, and Table S2, which is a summary of laboratory test scores for LN patients. The file also contains Figure S1, which includes the receiver operator characteristic curves for citrate, hippurate and taurine. [file ar3530-S1.DOC]

Supplementary material for:

**Identification of Urinary Metabolites That Distinguish Membranous Lupus Nephritis From Proliferative Lupus Nephritis and Focal Segmental Glomerulosclerosis**

Lindsey E. Romick-Rosendale^1^, Hermine I. Brunner^2^, Michael R. Bennett^3^, Rina Mina^4^, Shannen Nelson^5^, Michelle Petri^6^, Adnan Kiani^7^, Prasad Devarajan^8^, and Michael A. Kennedy*

**Table S1. Summary of classifications from histological analyses of kidney biopsy samples**

**Table S2. Summary of laboratory test scores for LN patients.**

**Figure S1. ROC curves for citrate, hippurate and taurine.**

| **Patients** | **INS/RPS Class** | **Mesangial expansion** | **Capillary proliferation** | **Cellular crescent** | **Fibrinoid necrosis** | **Wire-loop** | **Fibrosis** | **Tubular atrophy** | **AI** | **CI** |
| --- | --- | --- | --- | --- | --- | --- | --- | --- | --- | --- |
| 40 | 4 | Present | Absent | Absent | Absent | Absent | Present | Present | 2 | 3 |
| 41 | 4 | Absent | Absent | Absent | Absent | Absent | Absent | Absent | 0 | 0 |
| 42 | 4 | Present | Absent | Absent | Absent | Absent | Absent | Absent | 0 | 0 |
| 43 | 4 | Present | Present | Absent | Absent | Present | Present | Present | 6 | 2 |
| 44 | 4 | Present | Present | Absent | Absent | Present | Present | Present | 8 | 3 |
| 45 | 4 | Present | Present | Present | Present | Present | Present | Present | 7 | 3 |
| 46 | 4 | Present | Absent | Absent | Present | Present | Present | Absent | 3 | 1 |
| 50 | 5 | Present | Absent | Absent | Absent | Absent | Present | Present | 2 | 1 |
| 51 | 5 | Absent | Absent | Absent | Absent | Absent | Present | Present | 0 | 2 |
| 52 | 5 | Present | Absent | Absent | Absent | Absent | Present | Present | 1 | 4 |
| 53 | 5 | Present | Absent | Absent | Absent | Absent | Present | Present | 2 | 5 |
| 54 | 5 | Present | Absent | Present | Present | Absent | Present | Present | 4 | 1 |
| 55 | 5 | Present | Absent | Absent | Absent | Absent | Present | Present | 2 | 1 |
| 56 | 5 | Present | Absent | Absent | Absent | Absent | Absent | Absent | 0 | 0 |

**Table S1. Summary of classifications from histological analyses of kidney biopsy samples**

| **Patients** | **Lag time between biopsy and sample collection^&^** | **Total SLEDAI Score** | **SLEDAI Renal Score** | **Extrarenal SLEDAI Score** | **Systolic blood pressure** | **Diasystolic blood pressure** | **Erythrocyte sediment-ation rate** | **Serum Ceratinine** | **Creatinine**  **clearance** |
| --- | --- | --- | --- | --- | --- | --- | --- | --- | --- |
| 40 | -48 | 14 | 8 | 6 | 123 | 80 | 23 | 0.7 | 123 |
| 41 | -55 | 4 | 4 | 0 | 97 | 66 | 21 | 0.2 | 112 |
| 42 | -66 | 14 | 12 | 2 | 115 | 62 | 78 | 0.6 | 151 |
| 43 | 12 | 4 | 0 | 4 | 128 | 73 | 124 | 0.6 | 98 |
| 44 | -13 | 16 | 12 | 4 | 140 | 83 | 6 | 0.8 | 135 |
| 45 | -38 | 0 | 0 | 0 | 153 | 83 | 70 | 0.8 | 70 |
| 46 | -29 | 12 | 8 | 4 | 113 | 69 | 41 | 0.7 | 126 |
| 50 | 18 | 6 | 4 | 2 | 97 | 63 | 97 | 0.8 | 86 |
| 51 | 0 | 20 | 12 | 8 | 140 | 72 | 65 | 0.9 | 101 |
| 52 | -12 | 6 | 4 | 2 | 109 | 67 | 22 | 0.8 | 124 |
| 53 | -17 | 8 | 4 | 4 | 99 | 64 | 45 | 2.0 | 51 |
| 54 | -19 | 12 | 8 | 4 | 114 | 69 | 65 | 0.8 | 118 |
| 55 | -47 | 4 | 4 | 0 | 106 | 57 | 30 | 0.6 | 184 |
| 56 | -57 | 8 | 8 | 0 | 148 | 69 | 20 | 0.8 | 142 |

**Table S2. Summary of laboratory test scores for LN patients.**

& negative values represent urine samples collected PRIOR to the date of kidney biopsy.

**Figure S1. ROC curves for citrate, hippurate and taurine.**

**Citrate**

**
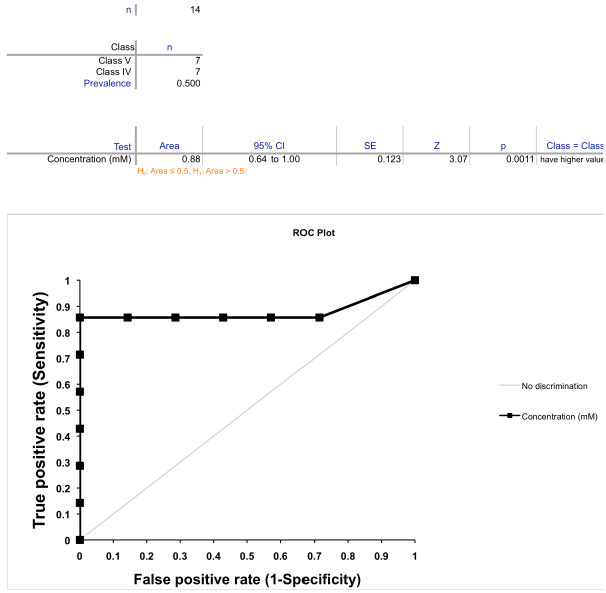
**

**Hippurate**

**
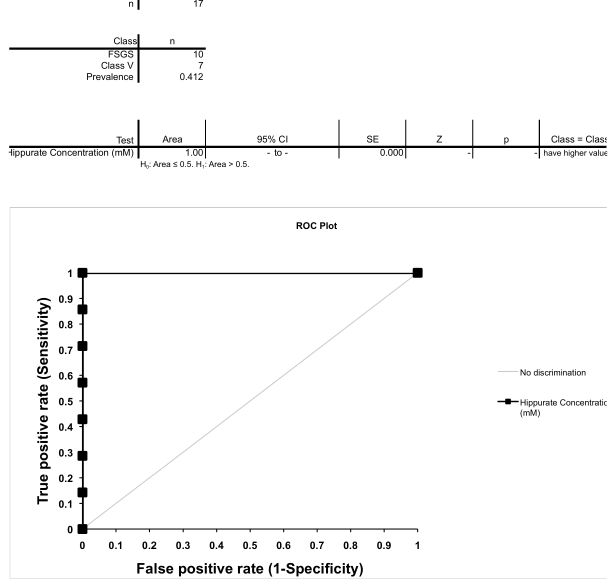
**

**Taurine**

**
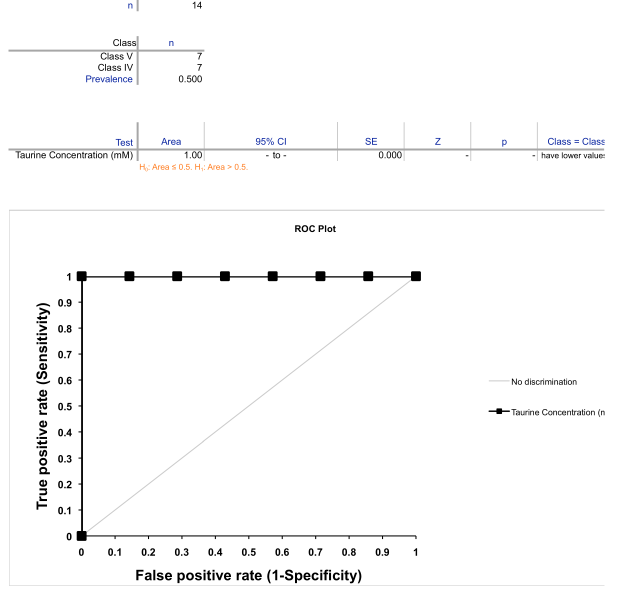
**
